# A Combination of Intrastromal and Intracameral Injections of Amphotericin B in the Treatment of Severe Fungal Keratitis

**DOI:** 10.1155/2016/3436415

**Published:** 2016-09-19

**Authors:** Jianzhang Hu, Jingjin Zhang, Yanling Li, Xiaoli Han, Weidong Zheng, Juan Yang, Guoxing Xu

**Affiliations:** The Eye Center of the First Affiliated Hospital of Fujian Medical University, Fujian Eye Institute, 20 Chazhong Road, Fuzhou 350005, China

## Abstract

*Purpose*. To evaluate the efficacy of a combination of intrastromal and intracameral injections of amphotericin B in the treatment of severe recalcitrant fungal keratitis.* Methods*. Patients with severe fungal keratitis who were resistant to conventional antifungal medical treatments and needed potential surgical intervention were recruited at the First Affiliated Hospital of Fujian Medical University between January 2012 and July 2013. The patients were treated with a combination of intrastromal and intracameral injections of amphotericin B (25 *μ*g/mL and 50 *μ*g/mL, resp.). Selectively repeated injections were performed as necessary. The efficacy, complications, and outcome were evaluated.* Results*. Nine patients (9 eyes) were involved in this study. All 9 cases responded favorably, and the clinical appearance of serious corneal damage and intraocular extension was resolved after the treatment. Four eyes required only 1 injection, and 5 eyes required repeated injections. Seven corneal ulcers healed with leucoma, and 2 healed with adherent leucoma. All of our cases had a marked increase in the anterior chamber reaction and pain immediately after the injection. There was no obvious clinical evidence of corneal or lenticular toxicity in any patient.* Conclusions*. A combination of intrastromal and intracameral injections of amphotericin B may be safe and effective for the treatment of severe fungal keratitis that is resistant to conventional therapy.

## 1. Introduction

Fungal keratitis is one of the major causes of blindness in developing agricultural countries and is usually difficult to treat [1, 2]. Some patients who live in remote and economically impoverished regions may delay visiting the hospital; some are underdiagnosed and inappropriately treated and often suffer serious consequences. The keratitis may aggravate and lead to serious complications such as corneal staphyloma, descemetocele, endophthalmitis, perforation, and blindness.

The most frequently isolated causes of fungal keratitis are* Fusarium* and* Aspergillus*, which are highly virulent microorganisms and are partially resistant to most antifungal medications [3]. The hypha is capable of penetrating the intact Descemet's membrane and rapidly entering the anterior chamber. In such cases, conventional treatment such as the use of antifungal medications, including fluconazole, topical natamycin, amphotericin B, or the combination with oral fluconazole, seems to obtain poor results. Moreover, the corneal penetration and bioavailability of many of the available topical antifungal preparations are suboptimal, making it difficult to treat cases of deep mycotic keratitis [4, 5]. Keratoplasty may be an effective way to control the fungal infection [6]. However, because it is not as effective as an optical keratoplasty performed on a quiescent eye after healing [7], and partly because of the limited and erratic supply of donor corneas in China, it seems wise to try to postpone keratoplasty until after healing.

To overcome these problems, investigators have evaluated alternate routes such as intracameral or intrastromal amphotericin B injections to treat fungal keratitis [8–11]. When used as a topical antifungal agent, amphotericin B has broad-spectrum antifungal activity but strong cytotoxicity at high concentrations and poor corneal penetration [12, 13]. In our clinical experience, intrastromal injections of amphotericin B often obtain poor results in the treatment of severe fungal keratitis when the hypha has invaded the anterior chamber and pupillary space. Additionally, intracameral injections of amphotericin B are not very effective at inhibiting the hypha growth in the stroma and usually cause some complications, including immediate anterior chamber reactions, secondary glaucoma, and cataract. In this study, we used a combination of intrastromal and intracameral injections of different concentrations of amphotericin B as an alternative to conventional therapies and evaluated its efficacy in the management of severe keratomycosis with serious corneal damage and intraocular extension which was resistant to conventional antifungal medical treatment and may have required potential surgical intervention.

## 2. Patients and Methods

### 2.1. Patients

From January 2012 through July 2013, 103 patients with fungal keratitis were hospitalized at the Eye Center at the First Affiliated Hospital of Fujian Medical University, Fujian Eye Institute, Fuzhou. As the largest referral eye center in Southeast China, this institution serves a large proportion of patients with eye diseases in Fujian province and in neighboring provinces. Institutional review board approval was obtained. Each of the patients gave informed consent for participation before treatment.

In this interventional case series, patients diagnosed of fungal keratitis involving serious corneal damage and visible fungal mass in the anterior chamber that were resistant to topical and/or systemic antifungal treatment underwent intrastromal combined with intracameral injections of amphotericin B at doses of 25 micrograms/0.1 mL and 50 micrograms/0.1 mL, respectively. The epidemiological characteristics, predisposing factors, clinical features, microbiological findings, treatment protocol, and final outcome data for each patient were collected.

### 2.2. Diagnostic Methods

The diagnosis of fungal keratitis was made on the basis of clinical evaluation, positive smear, and fungal cultures. The corneal scrapings were obtained aseptically from the leading edge or base of the ulcer. A portion of each scraping was examined microscopically for the presence of fungi by staining with 10% potassium hydroxide (KOH) or Gram stain. Another portion was subjected to fungal culture following inoculation onto Sabouraud glucose agar (SGA) and strain identification.

### 2.3. Initial Medical Treatment

Once the diagnosis of fungal keratitis was established, medical treatment with antifungal agents was initiated. Patients received 0.5% fluconazole every half hour, combined with 5% natamycin or 0.25% amphotericin B every two hours. The patients were also treated with 200 mg of oral itraconazole daily for 21 days. Those with hypopyon received an intravenous injection of fluconazole (100 mg) twice daily and atropine drops once daily. If the infection was not controlled or continued to deteriorate with intensive antifungal therapy, intrastromal or intracameral amphotericin B injection, intrastromal combined with intracameral amphotericin B injection, or surgical intervention was performed according to the state of infection.

### 2.4. Inclusion Criteria

Combined intrastromal and intracameral injection of amphotericin B was recommended if the infection was not controlled with 1 week of the antifungal therapy described above, showed a tendency to aggravation, and presented with serious corneal damage and intraocular extension, such as severe corneal inflammation, diffuse edema and opacity, local staphyloma and descemetocele (an ulcer reaching Descemet's membrane), increasing endothelial plaque and hypopyon, or visible fungal mass in the anterior chamber and pupillary space.

Initially, we considered this form of management when a severe keratomycosis failed to respond to medical treatment, a donor cornea was not immediately available, and evisceration was most likely needed. After our successful treatment of the first case, and with our experience of single intrastromal or intracameral injection, we believed that a combination of intrastromal and intracameral injections might be beneficial and tried it in additional cases. Therapeutic keratoplasty was considered the next option if donor corneas were available.

### 2.5. Exclusion Criteria

Cases that had some involvement of adjacent sclera, frank corneal perforation, shallow anterior chamber, and presence of intravitreal fungal mass by B-ultrasound scanning were excluded from this management.

### 2.6. Injection Procedure

Amphotericin B was obtained in pure powder form and was reconstituted in 5% dextrose to obtain concentrations of 25 *μ*g/mL and 50 *μ*g/mL. All the injection procedure was carried out by Dr. Jianzhang Hu using an operating microscope after administering peribulbar and topical anesthesia under aseptic conditions.

Regarding intrastromal injection, with the bevel down, a 27-gauge needle was inserted obliquely from the uninvolved, clear area to reach just flush with the abscess at the mid-stromal level (intended level for drug deposit) in each case. Amphotericin B (25 *μ*g/mL) was injected in 4–6 divided doses around the ulcer to form a drug deposit around the circumference of the lesion. The total amount of drug injected intrastromally ranged from 0.05 mL to 0.1 mL.

As for intracameral injection, a limbal incision was made with number 11 surgical blade at the clear corneal sides, and the endothelial plaque region, hypopyon, and fungal mass were gently aspirated and subsequently inoculated on SGA. A volume of 0.5 *μ*g amphotericin B (50 *μ*g/mL) in 0.1 mL was intracamerally injected using a 30-gauge needle on a tuberculin syringe.

### 2.7. Treatment and Evaluation after Injection

Selectively repeated intrastromal or intracameral or combined intrastromal and intracameral injections were performed as necessary on the basis of the clinical response. If there was an aggravating inflammation, edema, opacity, or ulcer in the cornea, repeated intrastromal injections were scheduled with an interval of more than 5 days. In addition, repeated intracameral injections were also scheduled until the endothelial plaque, hypopyon, and fungal mass in the anterior chamber disappeared or until the treatment was deemed to have failed. The interval between repeat intracameral injections was more than 3 days.

Topical natamycin, fluconazole, and atropine were continued along with the injections. All patients were evaluated daily, including visual acuity, intraocular pressure, complications, and situation of ocular infection. Follow-up ranged from 2 to 4 months. Treatment success was defined as resolution of the corneal infiltrate, disappearance of the anterior chamber inflammation, and healing of the epithelial defect.

## 3. Results

Nine patients (9 eyes) were involved in this study. Of the 9 patients in this study, the mean age at presentation was 55.22 ± 8.6 years (range, 37 to 71 years). There were 6 males and 3 females. Five patients were farm workers, 2 patients were physical laborers, and 2 patients were temporary employees. The mean age of duration from the onset of symptoms to presentation at our institution ranged from 17 to 63 days (means, 39.22 ± 10.45 days). The risk factors identified in these cases were corneal trauma with vegetable matter (5 eyes), contact lens use (1 eye), and unknown factors (3 eyes). Three patients had a history of diabetes mellitus, and 4 patients had previously received topical steroid therapy (Table 1).

Before injection, initial visual acuity was counting fingers or worse. The size of the ulcers varied from 4.0 to 8.0 mm, and the size of the infiltrate varied from 5.0 to 9.0 mm. In all 9 eyes, laboratory test results before injection showed a positive stain and/or culture for the presence of fungus. Of the 7 eyes with positive cultures, 3 scrapings grew* Fusarium* spp., 2 grew* Aspergillus* spp., 1 was identified as* Alternaria species*, and there was 1 unidentified species (Table 1). The aspirate from the anterior chambers of 8 eyes contained fungal elements.

### 3.1. Progress of Treatment

Four eyes healed after just 1 treatment with combined intrastromal and intracameral injections (Iii), and 5 eyes needed subsequent injections. Of the 4 eyes that required only 1 treatment, the inflammatory response was observed to weaken after 2 days, and hypopyon disappeared between 3 and 10 days after injection (mean, 6.17 ± 2.27 days). Of the 5 eyes that required more than 1 treatment, 1 eye required one additional combined intrastromal and intracameral injection, 1 eye required another 1 intrastromal injection, 2 eyes required another 2 intracameral injections, and 1 eye required another 2 intracameral injections and 1 intrastromal injection in succession (case 5; Table 1). Case 5, which was identified as* Aspergillus* spp. infection, responded well after the first injection, but the cornea showed no obvious improvement, and hypopyon and fungal mass showed slight increases on day 5. Therefore, the patient received 2 intracameral injections on day 7 and day 12 and 1 intrastromal injection on day 15, and the infection was controlled 7 days after the last intervention.

### 3.2. Complications

In each of the 9 patients, the procedure was performed successfully, and no severe intraoperative or postoperative complications were observed. In 2 patients (cases 2 and 3), there was minimal intrastromal bleeding in the inferior part of the cornea, but this resolved in 5–7 days. All patients complained of slight pain immediately after injection, and 4 patients reported a significant increase in pain following the injections. Marked uveitis was observed in all eyes with exudative membrane in the anterior chamber the day after the first injection, which decreased by the second day. Secondary glaucoma occurred in 6 eyes the day following every intracameral injection, especially in patients who received several intracameral injections, and the intraocular pressure was lowered by intravenous infusion of mannitol and application of Timolol Maleate drops twice daily.

### 3.3. Outcome of Treatment

In all 9 eyes, the clinical appearance of fungal invasion, including corneal infiltration, hypopyon, endothelial plaque, and fungal mass in the anterior chamber, resolved after the treatment. Seven corneal ulcers healed with leucoma, and 2 healed with adherent leucoma. Final visual acuity improved to hand movement to 20/40 depending on the location of the remaining scar (Table 1). The time from the first injection to complete resolution of the infection ranged from 18 to 53 days. None of the eyes exhibited vitreous opacity or band formation, and there was no evidence of either local or systemic toxic side effects. There was no recurrence of the infection after withdrawal of all of the antifungal agents. All patients ultimately developed a cataract.

## 4. Discussion

The primary treatment of fungal keratitis is still the use of antifungal medications, including topical natamycin, amphotericin B, or fluconazole, alone or combined with oral antifungal medications. This approach seems to be effective in the early stages of the disorder. The antifungal agents currently used for fungal keratitis possess a narrow spectrum of activity, toxicity, and lack of effective penetration into deeper layers of the cornea [4, 5]. In recent years, it was still much difficult to treat severe keratitis caused by antimycotic-resistant fungi, even though there was great development of new broad-spectrum antimycotics. To overcome these problems, attempts at site-directed drug deposition have been made in the form of intracameral injections, intrastromal injections, intravitreal injections, and posterior Sub-Tenon injections [8–11, 14–16].

Severe corneal infections may result in extensive corneal melting, acute perforation, endophthalmitis, and rapid visual loss [17]. Meanwhile, the hypha may be penetrating the intact Descemet's membrane and rapidly invading the anterior chamber. At this point, oral and/or intravenous and/or topical drops of antifungal agents obtain poor results, just as in cases reported here. In order to achieve adequate intracorneal concentrations of antifungals, intrastromal injections of antifungals have been tried [8, 14–16]. This treatment was used in infections which were mostly focused in the cornea and seldom invaded the anterior chamber. Additionally, to achieve adequate drug levels in the anterior chamber, subconjunctival and intraocular injections have been tried [9–11, 18]. Subconjunctival injections can produce long-standing periocular inflammation and can lead to epithelial ulcerations, with little penetration into the aqueous [18]. From clinical experience, intracameral injections may achieve therapeutic concentrations in the aqueous, but they are suboptimal in the corneal stroma, where fungal invasion can easily lead to corneal perforation. Hence, in this study, a combination of intrastromal and intracameral injections was administered and successfully treated 9 severe cases without surgical intervention.

Amphotericin B has been shown to be effective in treating systemic mycosis caused by natamycin-resistant filamentous fungi [5, 19]. It has a wide spectrum of activity but has cytotoxicity and poor penetration [12, 13]. It remains to be a potent agent in the treatment of severe keratomycosis, and its efficacy is closely dependent on the ability to achieve optimal drug levels in the cornea [5, 19]. So, to improve the efficacy of amphotericin B, selecting a proper formulation and mode of application is the key [10].

In our series, none of the 9 severe cases had responded to topical antifungal therapy, and we therefore decided to proceed with combined intrastromal and intracameral drug delivery. Successful administration means a more focused concentration of amphotericin B on the side of infection, as well as more reduction of tissue damage. In our opinion, successful injection includes the following aspects. First, the drug should be injected around the abscess on the cornea to form a deposit and create a barrier around the circumference of the ulcer. Second, the needle should be inserted from the uninvolved area to reach just flush with the abscess. Third, to avoid piercing the cornea during intrastromal injection, the needle should be beveled down and inserted to the mid-stromal level slowly and accurately. Fourth, during intracameral injection, when the needle passes through the endothelium and reaches the anterior chamber without resistance, the clinician should stop advancing and begin injecting.

The ideal dose of amphotericin B for intrastromal and/or intracameral use is undetermined, but it should achieve maximum therapeutic effect with minimal side effects. Study has shown that intrastromal injection of amphotericin B at a concentration of less than 10 *μ*g per 0.1 mL is safe in rabbit corneas [20], and at a concentration of 5 *μ*g per 0.1 mL it does not appear to be deleterious to keratocytes or endothelial cells in the clinic [8]. Up to 50 *μ*g of intracamerally injected amphotericin B is also well tolerated by rabbit eyes, and it causes only reversible iritis and clouding of the lens [21]. Some reports showed that the clinical dose recommended for intracameral injections of amphotericin B is 10 to 30 *μ*g in 0.1 to 0.2 mL [9–11]. In our cases, up to 2.5 *μ*g of amphotericin B was intrastromally injected and up to 5.0 *μ*g was intracamerally injected, and a therapeutic concentration was readily delivered without significant adverse effects. The reason that the concentration of amphotericin B used for intrastromal injection (25 *μ*g/mL) was lower than that for intracameral injection (50 *μ*g/mL) is that, first, lower concentrations cause less cornea toxicity and damage. Otherwise, for these severe cases, high concentrations of amphotericin B may easily have led to cornea perforation. Second, amphotericin B in the anterior chamber may penetrate the endothelium into the stroma and gradually increase the concentration of amphotericin B in the stroma to achieve a therapeutic effect. Although this option was not attempted, our study has shown that this treatment was very effective.

Several studies reported the use of intracameral or intrastromal amphotericin B injection in the treatment of fungal keratitis and found that all patients responded favorably with complete clearing of corneal infection and hypopyon and no evidence of corneal or lenticular toxicity [8–11]. In our study, we also found that injections of amphotericin B were safe. None of our patients developed systemic toxic effects from the drug after the injection. No corneal decompensation was shown. Animal studies have shown that low doses of intracameral amphotericin B injection could cause reversible iritis and clouding of the lens [21]; however, a recent similar preliminary study did not report any immediate increase in pain or inflammation [10]. We speculated that complications may be related to the severity of the fungal infection. In this study, all 9 cases involved serious infection. All of our cases had marked increases in the anterior chamber reaction and pain immediately after injection, consistent with a report by Kuriakose et al. [11]. The pain mostly resolved in 12 hours and was presumed to be related to the stimulation of the ciliary body by amphotericin B. The anterior chamber reaction accompanied by exudative membrane was speculated to be caused by three factors: one was the toxic effect of amphotericin B on the iris-ciliary body; one was the stimulation of the iris-ciliary body by the degradation products of fungus decomposed by the drug; and one may have been exudation from the inflamed dilated iris vessels secondary to the decompression caused by the procedure. The anterior chamber reaction may also result in a transient increase of intraocular pressure, which is mainly caused by obstruction of aqueous humor drainage.

Case 5 was the most severe case in this study; he received 2 intrastromal and 3 intracameral injections, and the infection was finally controlled. In our clinical experience, a total of 3 intrastromal and/or 3 intracameral injections may be generally sufficient for the management of severe keratomycosis, thus avoiding complications related to possible toxicity which might result from increased doses of amphotericin B. In addition, the interval between repeat injections should be sufficient to allow adequate healing time of wounds caused by the procedure. We thought that intervals of more than 5 and 3 days between repeat intracameral and intrastromal injections, respectively, were appropriate.

The keratitis in the patients in this study was so severe that they needed potential surgical interventions such as keratoplasty, or even evisceration, in our experience. Our treatment allows the clinician to avoid keratoplasty as a primary mode of treatment, and avoidance of this surgery was beneficial on several counts. However, our treatment only resolved the infection at an acute stage of fungal keratitis and saved the eyeball, but it could not improve the patients' visual acuity. Most of the corneal ulcers healed with leucoma and had extensive corneal scarring after resolution of the infection. This outcome was also common for serious corneal damage. All patients ultimately developed a cataract, which was most likely related to an inflammatory reaction, amphotericin B toxicity, and/or injection trauma. Thus, optical keratoplasty and cataract extraction should be considered the next option.

Of the cases in this study, the mean duration from the onset of symptoms to presentation at our institution was 39.2 ± 10.5 days, much longer than that in a previous report of a spectrum of fungal keratitis in North China which was 26.6 ± 19.0 days. There, 5 eyes still required more than 1 injection for successful resolution of the infection. The patients who needed repeated injections had a preexisting history of diabetes mellitus (1 case) or previous topical steroid therapy (4 cases). It was reported that steroids may increase disease severity and may delay fungal clearance [22, 23]. It seems that a long disease course, a history of diabetes mellitus, and previous topical steroid therapy may aggravate the keratitis and increase the difficulty of injection therapy. It was reported that* Aspergillus* spp. have a stronger virulence than* Fusarium* spp. and* Alternaria species*  [24]. In our study, the cases identified as* Aspergillus* spp. infection all needed repeated injections, such as cases 5 and 7. Therefore, an understanding of these potential risk factors may provide ophthalmologists with valuable information before injection and help properly evaluate the treatment.

In summary, the results from this study showed that a combination of intrastromal and intracameral injections of amphotericin B was safe and effective in the treatment of severe fungal keratitis that was resistant to conventional therapy. Different concentrations of amphotericin B can be used in the intrastromal and intracameral injections. Repeated injections may be necessary in some cases. Adept injection skills and correct recognition of complications and potential risk factors are important for successful treatment.

## Figures and Tables

**Table 1:** Clinical data of patients with severe fungal keratitis treated with combined intrastromal and intracameral amphotericin B.

Case number	Age (years)/gender	Risk factor for fungal keratitis	History	Duration of onset of symptoms (days)	Initial BCVA	Size of ulcer (mm)	Fungal identification	Repeated injection	Final BCVA	Healed with
1	37/F	Corneal trauma	Diabetes	27	CF	5 × 6	*Fusarium*	1 Isi	20/40	Leucoma
2	56/M	Unknown	Steroid	17	HM	5.5 × 6.5	Not identified	2 Ici	20/200	Leucoma
3	67/M	Corneal trauma	Diabetes	35	LP	6 × 7	*Alternaria*	1 Iii	CF	Leucoma
4	71/M	Unknown	No	33	HM	6 × 6	*Fusarium*	—	CF	Leucoma
5	57/F	Corneal trauma	Steroid	39	HM	7 × 8	*Aspergillus*	2 Isi + 1 Isi	HM	Adherent leucoma
6	63/F	Contact lens	Diabetes	46	LP	6 × 7	Negative	—	20/300	Leucoma
7	56/M	Corneal trauma	Steroid	63	LP	5 × 7	*Aspergillus*	2 Ici	HM	Adherent leucoma
8	47/M	Unknown	No	52	HM	5.5 × 6.5	*Fusarium*	—	CF	Leucoma
9	43/M	Corneal trauma	Steroid	41	CF	6 × 8	Negative	—	20/200	Leucoma

CF, counting fingers; F, female; HM, hand movements; Iii, combined intrastromal and intracameral injections; Isi, intrastromal injections; Ici, intracameral injections; LP, light perception; M, male.
